# Laser Microdissection of Sensory Organ Precursor Cells of *Drosophila* Microchaetes

**DOI:** 10.1371/journal.pone.0009285

**Published:** 2010-02-19

**Authors:** Eulalie Buffin, Michel Gho

**Affiliations:** 1 Université Pierre et Marie Curie-Paris 6, UMR 7622, Paris, France; 2 CNRS, UMR 7622, Paris, France; National Institutes of Health (NIH), United States of America

## Abstract

**Background:**

In *Drosophila*, each external sensory organ originates from the division of a unique precursor cell (the sensory organ precursor cell or SOP). Each SOP is specified from a cluster of equivalent cells, called a proneural cluster, all of them competent to become SOP. Although, it is well known how SOP cells are selected from proneural clusters, little is known about the downstream genes that are regulated during SOP fate specification.

**Methodology/Principal Findings:**

In order to better understand the mechanism involved in the specification of these precursor cells, we combined laser microdissection, toisolate SOP cells, with transcriptome analysis, to study their RNA profile. Using this procedure, we found that genes that exhibit a 2-fold or greater expression in SOPs versus epithelial cells were mainly associated with Gene Ontology (GO) terms related with cell fate determination and sensory organ specification. Furthermore, we found that several genes such as *pebbled/hindsight, scabrous, miranda, senseless*, or *cut*, known to be expressed in SOP cells by independent procedures, are particularly detected in laser microdissected SOP cells rather than in epithelial cells.

**Conclusions/Significance:**

These results confirm the feasibility and the specificity of our laser microdissection based procedure. We anticipate that this analysis will give new insight into the selection and specification of neural precursor cells.

## Introduction

In *Drosophila*, the small external sensory organs (microchaetes) located on the dorsal part of the thorax has become an excellent system to analyse mechanisms involved in the acquisition and maintenance of neural precursor cell identity from a non-differentiated state [1], [2]. In this system, each sensory organ develops from a single SOP that arises from a cluster of equivalent cells called proneural cluster. Cells of a proneural cluster are defined by the expression of the proneural genes *achaete* and *scute (ac/sc)* that provide them with the competence to become SOP [3], [4]. In each cluster, the proneural competence is progressively restricted to only one cell that accumulates the highest level of proneural proteins and that will become the SOP whereas the others cells remain epithelial cells. This process of SOP selection depends on both the auto and cross regulation of proneural gene expression [4] and the activation of the Notch signalling pathway. This later involves cell-cell interactions mediated by the Notch receptor and it's ligand Delta in such a way that in each cluster, one cell (the future SOP) will express higher level of the ligand Delta and will activate Notch receptors in neighbouring (future epithelial) cells [5], [6]. Notch signalling promotes the transcription of *Enhancer of split complex* genes that repress proneural gene expression and prevents the acquisition of neural fate [4], [7], [8].

Despite considerable progress in our knowledge of the mechanisms underlying SOP selection, relatively few downstream target genes regulated by this proneural regulatory network are known. The gene *senseless* (*sens*), which encodes a zinc finger transcription factor, is one of the known downstream genes. Sens is expressed in SOPs and has been shown to act as a binary switch in the proneural cluster. High levels of Sens up regulate *ac/sc* expression and, conversely, low levels repress *ac/sc* expression [8], [9]. It is tempting to speculate that other Sens-like factors remain to be discovered.

In order to determine the genome-wide response associated with SOP fate acquisition, we propose an original protocol that combines laser microdissection, to isolate individually SOPs from epithelial cells, and transcriptome analysis, to compare the RNA profiles of SOPs cells from that of their sibling epithelial cells. Our analysis revealed that genes exhibiting a two-fold or greater expression in SOPs were mainly associated with gene ontology (GO) term related to sensory organ specification and neurogenesis. Moreover, from this set of genes, almost twenty genes were previously found to be expressed in SOPs. These data show the feasibility and the specificity of the laser microdissection technique in isolating identified cells from this type of system. We anticipate that this approach will give new insights into the selection and specification of neural precursor cells. Furthermore, we believe that this technique can easily be extended to different epithelia and as such will be useful in investigating specific cell transcriptomes.

## Materials and Methods

### Fly Stocks

The *neuralized^p72^*-Gal4 driver was used to express in pI cells the construction mCD8::GFP using the UAS/Gal4 system [10]. *neur*>mCD8::GFP flies were reared on a standard *Drosophila* diet. White pupae were selected and maintained at 25°C prior dissection.

### Fixation and Mounting

The notum from *neur*>mCD8::GFP pupae were dissected out in PBS and fixed in cold absolute ethanol for 10 minutes. A fixation longer than 15 min, hardes the tissue and makes the microdissection difficult. Three to five fixed nota were transferred directly from ethanol with a Pasteur pipette and then carefully flattened and dried with the epithelium facing down on a thermolabile membrane slides for laser microdissection (See Fig S1).

### Laser Microdissection

Laser microdissection was realized on a MMI cellcut microdissection system coupled to an Eclipse TE-2000 inverted fluorescent microscope (Nikon Instrument). The parameters used were: focus 40, speed 1, power 74 at objective 60X and 4 to 8 laser rounds were required to cut through a notum. Selected areas were cut from the tissue by an UV laser beam. To keep the SOP integrity and preserve RNA from the heat of the laser, we took care to leave a space between the laser circle and the cell limit (around 5 µm).

### RNA Extraction and Amplification

Total RNA was extracted from microdissected cells by using the picopure RNA isolation kit (Molecular devices - Arcturus) following manufactures instructions with minor modifications as described below. We incubated the tubes containing microdissected cells with 20 µl of extraction buffer at 42°C upside down for 30 min. Then, after centrifugation, the extracts pooled were passed through a single RNA purification column. During purification, we treated the column with DNAse I (Qiagen) for 30 min at room temperature to avoid genomic DNA contamination. We obtained 0,1–0,5 µg of total RNA from a sample of 1000 microdissected cells.

After extraction, RNA was amplified by using the MessageAmp II aRNA Amplification Kit (Ambion). We proceeded with two rounds (9 h each) of *in vitro* transcription. After each round, the RNA purification column was treated by DNAse I (Qiagen) for 10 min at room temperature before aRNA elution. For better RNA integrity, we carried out all the amplification processes in one step directly after RNA extraction to avoid freezing the sample. Indeed, in addition to the usual recommendations about manipulating RNA, we avoided, as much as possible, freezing both the tissue before microdissection and the RNA samples between extraction and amplification. After two rounds of amplification, we obtained 20–50 µg of aRNA from a sample of 1000 microdissected cells.

For microarray hybridizations, UTP-amino allyls were integrated during the second round of *in vitro* transcription, for subsequent labelling with dyes Cy3 or Cy5 (Amino Allyl MessageAmp II kit - Ambion).

### qRT-PCR

We performed reverse transcription on 1 µg of aRNA using random primers from Roche and the SuperscriptII reverse transcriptase from Invitrogen. The same quantity of cDNA (50–100 ng) from SOPs or epithelial cells was then used to perform semi quantitative PCR (30 cycles) or qRT-PCR for several genes.

qRT-PCR was performed on Bio-Rad iCycler IQ™ using SYBR green PCR master mix with the following parameters: 95°C-3 min followed by 40 cycles of 95°C-30 sec, 60°C-30 sec and 72°C-30 sec. Quantifications were made using the relative standard curve method. The standard curves were created by a series of 5 dilutions of cDNA synthesized from aRNA, extracted and amplified from 20 whole nota dissected and fixed as described here. Each dilution of the standard curves was amplified in duplicate and each sample of interest was amplified in triplicate. Curves of one experiment are shown in figure S2. mRNA levels detected by qRT-PCR were normalized to mRNA level of *taf11* used as reference gene.

### Microarray

Amplified and differently labelled aRNA from 1000 microdissected SOPs and an equivalent surface of epithelial cells were hybridized to INDAC Drosophila GeneChips (platform Montpellier GenomiX, Institut de Génomique Fontionnelle, UMR 5203 CNRS – U661 INSERM, Montpellier, France). Normalization of raw data was performed by LIMMA. The flagged spots and controls were removed from the analysis. No background correction was performed before normalization. Lowess normalization was used to normalize the M values for each array separately (within-array normalization). Genes exhibiting a signal ratio SOPs/epithelial cells superior than two were considered as SOPs-overexpressed genes for subsequent analysis. Gene Ontology analysis was performed with Flymine [11] that provides enriched GO terms ranked by significance. P values were calculated following a hypergeometric distribution (with Bonferroni correction).

### Data Deposition

The raw data associated with this manuscript are available on the Gene Expression Omnibus (GEO) according to MIAME standards under the following accession number: GSE18615.

## Results

### Purification of SOPs by Laser Microdissection

In order to identify SOP cells, we specifically expressed the construction mCD8::GFP to label SOP membranes and their progeny by using the Gal4/UAS expression system and the specific driver line neuralized^p72^Gal4 (neur>)[11]. The dorsal epithelium (or notum) of neu>mCD8::GFP pupae at 16h after puparium formation (APF) was dissected and fixed in ethanol. At this developmental time, most of the SOPs have not yet divided [12], [13].

**Figure 1:**
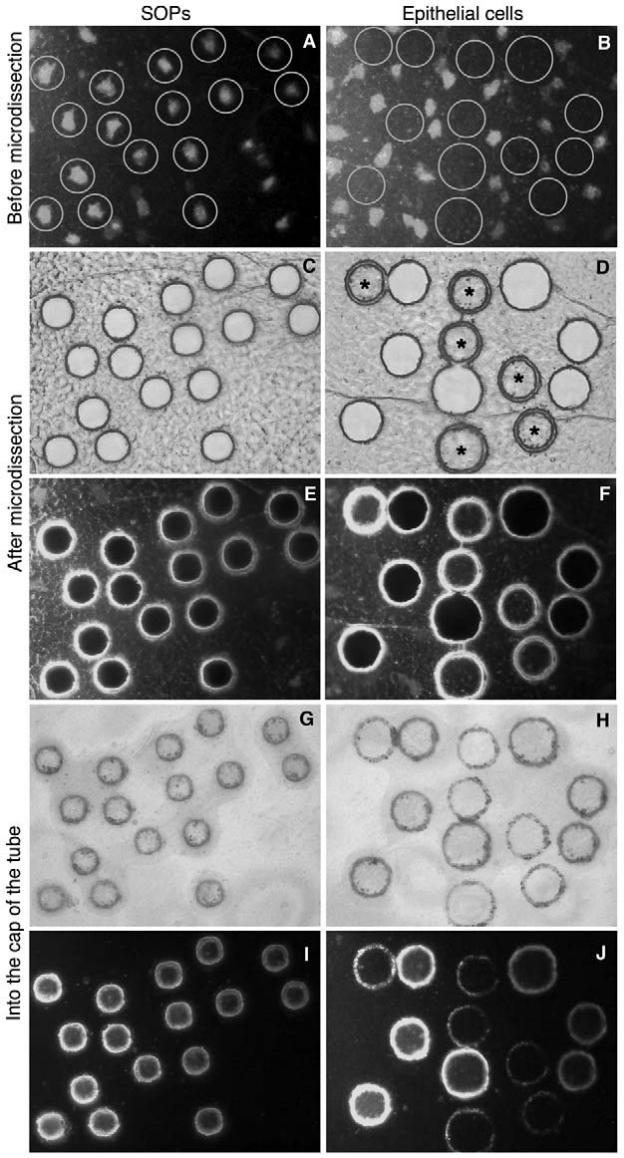
Laser Microdissection. Laser Microdissection of SOP cells (left column) and epithelial cells (right column). Fixed nota from *neur*>mCD8::GFP flies (16 h APF) that express GFP specifically in SOP cells. SOP cells were laser-cut following a circle pathway centered on each SOP (A). After cut, gaps corresponding to each SOP encircled remained on the nota (transmitted light in C and fluorescent light in E). In contrast, the captured SOP cells stuck to the lid of a microtube (transmitted light in G and fluorescent light in I). A similar procedure is shown for epithelial cell capture. These cells were isolated from areas without fluorescent SOP cells (B). Note that sometimes for SOP (not shown) as well as epithelial cell microdissection (asterisks in D) some areas were not captured and remain on the nota. Note also that, the fluorescence level was strongly reduced after laser beam application (I and J).

After mounting on a membrane slide, SOPs expressing GFP were identified by fluorescence and circled manually with a circle radius of 9 µm (Fig. 1A). Microdissected cells were then collected on an adhesive lid of a microtube placed onto the area (Fig. 1G–J). The success of the cell capture was visually confirmed by the gaps in the tissue after lid removal (Fig. 1C–F). We collected around 20 cells per notum, 20–50 cells on a cap and pooled around 20–50 tubes to proceed to the RNA extraction. Altogether, we collected around 1000 SOPs from 50 nota. In parallel, we captured tissue free of SOP fluorescent cells corresponding to epithelial cells (Fig. 1B). A similar integrated surface (around 250 000 µm^2^) was collected in order to standardize both samples.

Once the required number of cells was been collected, total RNA was extracted and amplified for analysis.

### Quantitative Real Time PCR and Microarray Data Confirm Differential Expression of Known Genes

**Figure 2:**
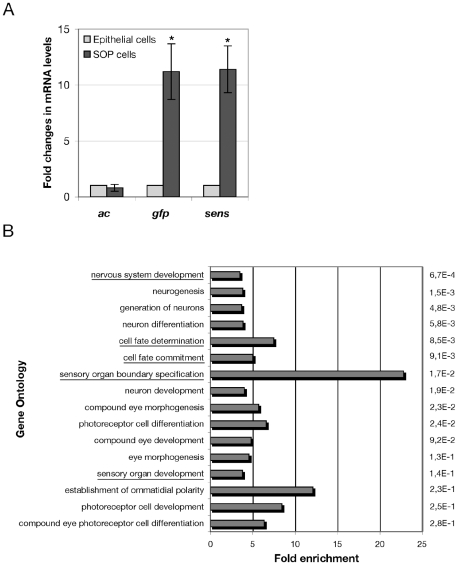
Microdissected SOP cells show specific gene expression and are enriched with genes associated with cell fate determination. (A) Fold changes represent the ratio between SOP and epithelial cell mRNA levels measured by qRT-PCR. Values obtained in epithelial cells were normalized to 1. Transcripts for *gfp* (ectopically expressed in SOP cells) as well *achaete (ac)* and *senseless (sens)* are shown. The mean and standard deviation of at least 3 independent experiments for each gene are represented. The difference between SOP and epithelial cell expression levels was considered significant when student test *P* value was inferior to 0,05 (indicated with asterisk). Note that, *achaete (ac)* expression was not different in SOPs and epithelial cells (ratio  = 0,8), whereas *gfp* and *senseless (sens)* were expressed 11,2 and 11,4 more times in SOPs than in epithelial cells respectively. (B) Genes overexpressed in SOPs (ratio of SOP/epithelial cell transcripts ≥2) were grouped according to their function on the basis of their ascribed GO terms. The 16 categories having lowest P-values with enrichment ≥3 are shown ranked. P-values (on the right) were calculated following a hypergeometric distribution (with Bonferroni correction). Fold enrichment was calculated as the ratio between the percentage of genes associated with a given GO term among SOP-over expressed genes and the percentage of genes associated with the same GO term throughout the entire genome. Note that many of the significant categories concern fate determination and nervous system (underlined).

We carried out reverse transcription following by PCR on some SOPs specific (*ac, sens*, [4], [9]) and non-specific (*rp49, taf11*) genes to verify the aRNA extracted and amplified from microdissected cells. Semi quantitative PCR (30 cycles) performed on the same quantity of cDNA from SOPs and epithelial cells showed that *Rp49, taf11* and *ac* seem to be expressed at similar levels in SOPs and epithelial cells. In contrast, as expected, *gfp* (that was ectopically expressed in SOPs) and *sens* were more highly expressed in SOPs than in epithelial cells (not shown). To verify these results, we performed quantitative real time PCR (qRT-PCR). We calculated the ratio of SOP/epithelial cells mRNA levels for each gene (Fig. 2A). Using this procedure, we confirmed that *ac* expression was not significantly different in SOPs and epithelial cells (ratio  = 0,8), whereas the expression of *gfp* and *sens* was higher in SOP than in epithelial cells (11,2 and 11,4 times respectively) (Fig. 2A and Fig. S2). The significant enrichment of transcripts corresponding to *sens* and *gfp* in SOPs confirms the usefulness and the specificity of aRNA material collected using the laser microdissection technique on fixed *Drosophila* nota.

Concomitantly to qRT-PCR analysis, we used DNA microarrays to identify genes differently expressed between microdissected SOPs and epithelial cells. This analysis revealed 127 genes whose expression was increased 2-fold or greater between SOPs and epithelial cells (Table 1). To analyse whether a particular biological process could be overrepresented in this data set, we regrouped the genes of this set according to their function that has been ascribed using Gene Ontology (Go) terms (www.geneontology.org). This analysis showed that 58% of these genes were associated with a specific function. Interestingly, 27% of this subset of genes were related to the nervous system. This category showed more than a three fold enrichment in the SOP-gene data set (Fig. 2B). More precisely, a hypergeometric test applied on this set of 127 genes, revealed a significant enrichment in GO terms related to nervous system development, sensory organ development and cell fate specification. Moreover, several eye photoreceptor cell development associated GO terms were also enriched in our SOP-gene data set (Fig 2B). Conversely, among GO terms that are significantly underrepresented and, as a consequence, enriched in their sibling epithelial cells, we found cuticle development and epithelium morphogenesis (data not shown). Furthermore, neither genes already known to belong to SOP-enriched genes nor genes associated with GO terms related with cell fate determination and sensory organ specification were found in this SOP non-enriched set of genes.

**Table 1:** Genes whose expression was increased 2-fold or greater between SOPs and epithelial cells.

	Flybase ID	Gene symbol	Gene name	SOPs/Epithelial cells signal ratio
1	FBgn0005561	sv	shaven	14,121155
2	FBgn0003053	peb	pebbled	13,55055
3	FBgn0019830	colt	congested-like trachea	10,6780015
4	FBgn0030396	CG2556		9,546415
5	FBgn0030589	CG9519		9,1127835
6	FBgn0052023	CG32023		7,569719
7	FBgn0037844	CG4570		7,20862
8	FBgn0052150	CG32150		6,548325
9	FBgn0002891	mus205	mutagen-sensitive 205	6,133705
10	FBgn0003326	sca	scabrous	6,06793
11	FBgn0005636	nvy	nervy	6,05058
12	FBgn0040842	CG15212		5,903055
13	FBgn0052392	CG32392		5,86501
14	FBgn0003995	vvl	ventral veins lacking	5,2886
15	FBgn0021776	mira	miranda	5,26211
16	FBgn0028536	CG15281		4,9381
17	FBgn0002573	sens	senseless	4,56789
18	FBgn0033772	CG12488		4,427495
19	FBgn0030432	CG4404		4,36984
20	FBgn0003996	w	white	3,8498
21	FBgn0034692	CG13502		3,582385
22	FBgn0033739	Dyb	Dystrobrevin-like	3,511521
23	FBgn0028537	CG31775		3,443735
24	FBgn0029839	CG4660		3,220815
25	FBgn0013725	phyl	phyllopod	3,114765
26	FBgn0028509	cenG1A	centaurin gamma 1A	3,11361
27	FBgn0053200	ventrally-expressed-protein-D		3,071915
28	FBgn0033507	CG12909		3,051625
29	FBgn0004779	Ccp84Ae		3,036835
30	FBgn0050118	CG30118		3,034455
31	FBgn0015393	hoip	hoi-polloi	2,982565
32	FBgn0036124	CG7839		2,97755
33	FBgn0036839	CG18136		2,9365975
34	FBgn0030027	CG1632		2,89352
35	FBgn0036137	CG7628		2,86596
36	FBgn0036369	CG10089		2,83541
37	FBgn0003187	qua	quail	2,827855
38	FBgn0030833	CG8915		2,8224225
39	FBgn0001090	bnb	bangles and beads	2,7679
40	FBgn0039154	CG6164		2,745795
41	FBgn0051523	CG31523		2,727035
42	FBgn0032871	CG2611		2,7265
43	FBgn0039118	CG10208		2,719125
44	FBgn0004511	dy	dusky	2,7177
45	FBgn0051800	CG31800		2,69935
46	FBgn0010383	Cyp18a1	Cytochrome P450-18a1	2,688865
47	FBgn0013765	cnn	centrosomin	2,67734
48	FBgn0058454	CR40454		2,6720405
49	FBgn0038318	CG6236		2,6341
50	FBgn0035878	CG7182		2,619305
51	FBgn0033275	CG14756		2,57977
52	FBgn0037723	SpdS	Spermidine Synthase	2,57505
53	FBgn0031273	CG2839		2,56041
54	FBgn0051352	CG31352		2,559855
55	FBgn0030001	CG15335		2,546375
56	FBgn0037240	Cont	Contactin	2,5257925
57	FBgn0039152	CG6129		2,52151
58	FBgn0002932	neur	neuralized	2,515405
59	FBgn0052827	CG32827		2,47839
60	FBgn0031764	CG9107		2,451235
61	FBgn0037137	Nopp140		2,450615
62	FBgn0019938	RpI1	RNA polymerase I subunit	2,44853
63	FBgn0003651	svp	seven up	2,439685
64	FBgn0034656	CG17922		2,43033
65	FBgn0038916	CG6560		2,4265
66	FBgn0039169	CG5669		2,42484
67	FBgn0039630	CG11843		2,386245
68	FBgn0002778	mnd	minidiscs	2,37934
69	FBgn0038120	CG10148		2,3619
70	FBgn0050349	CG30349		2,345675
71	FBgn0039335	CG5127		2,337975
72	FBgn0029568	CG11381		2,3251455
73	FBgn0004198	ct	cut	2,319
74	FBgn0010105	comm	commissureless	2,312085
75	FBgn0035521	CG1268		2,299415
76	FBgn0050007	CG30007		2,299075
77	FBgn0034224	CG6520		2,29819
78	FBgn0031706	nmr2	neuromancer2	2,27797
79	FBgn0037314	CG12000		2,271605
80	FBgn0000409	Cyt-c-p	Cytochrome c proximal	2,267055
81	FBgn0031604	CG15433		2,26653
82	FBgn0039404	CG14543		2,261375
83	FBgn0027903	CG12018		2,25671
84	FBgn0028855	CG15282		2,23759
85	FBgn0035532	CG15014		2,222355
86	FBgn0034528	CG11180		2,21393
87	FBgn0033802	CG17724		2,20619
88	FBgn0030958	CG6900		2,20591
89	FBgn0038017	CG4115		2,194645
90	FBgn0026378	Rep	Rab escort protein	2,173765
91	FBgn0028510	CG15261		2,173175
92	FBgn0052344	CG32344		2,163085
93	FBgn0031434	insv	insensitive	2,159285
94	FBgn0039563	CG4951		2,15345
95	FBgn0015907	bl	bancal	2,152305
96	FBgn0011638	La	La autoantigen-like	2,150125
97	FBgn0032297	CG17124		2,142305
98	FBgn0039271	CG11839		2,13788
99	FBgn0036043	CG8177		2,136985
100	FBgn0000340	cno	canoe	2,136715
101	FBgn0039829	CG15561		2,13596
102	FBgn0042092	CG13773		2,123165
103	FBgn0036096	CG8003		2,120365
104	FBgn0052645	CG32645		2,11955
105	FBgn0041004	CG17715		2,112665
106	FBgn0002563	Lsp1β	Larval serum protein 1 beta	2,10427
107	FBgn0029761	SK	small conductance calcium-activated potassium channel	2,09556
108	FBgn0052677	CG32677		2,071425
109	FBgn0005630	lola	longitudinals lacking	2,068285
110	FBgn0037248	CG9809		2,064895
111	FBgn0004551	Ca-P60A	Calcium ATPase at 60A	2,06413
112	FBgn0030501	BthD	BthD selenoprotein	2,063545
113	FBgn0023214	edl	ETS-domain lacking	2,05935
114	FBgn0015558	tty	tweety	2,05836
115	FBgn0003890	βTub97EF	beta-Tubulin at 97EF	2,05672
116	FBgn0050080	CG30080		2,054815
117	FBgn0038640	CG7706		2,05049
118	FBgn0030345	CG1847		2,041705
119	FBgn0046704	Liprin-α		2,03972
120	FBgn0039685	Obp99b	Odorant-binding protein 99b	2,03933
121	FBgn0029704	CG2982		2,03666
122	FBgn0036460	CG5114		2,03641
123	FBgn0026015	Top3β	Topoisomerase 3beta	2,032305
124	FBgn0036133	CG7638		2,022935
125	FBgn0033942	CG10112		2,01533
126	FBgn0036569	CG5414		2,014675
127	FBgn0024734	PRL-1		2,013035

Our data set of SOP-specific genes includes 19 known genes that have already been shown to be expressed in SOPs or involved in sensory organ development related mechanisms (Table 2). Among them, we found *sens*, confirming our qRT-PCR analysis, and other SOP-specific genes such as *cut (ct), neuralized (neur)* and *phyllopod (phyl)* [14]–[16]. We can also note *pebbled/hindsight (peb)* and *seven up (svp)* that are involved in photoreceptor development [17], [18], *scabrous (sca)* that plays a role in lateral inhibition processes via the regulation of Notch activity [19], and *miranda (mira)* involved in neuroblast and SOP asymmetric divisions [20], [21]. Moreover, among these genes, eleven have already emerged from microarray analysis performed on proneural clusters by Reeves and Posakony [22]. In addition to the well characterised genes such as *mira*, *peb*, *neur* or *phyl*, we can cite as an example of new SOP genes, *quail and insensitive* (Table 2). In contrast, some genes in our data set didn't appear in Reeves and Posakony microarray results. The most relevant examples are *sens* and *ct*, two well known SOP-specific genes [8], [14]. We can also cite *shaven, sca* or *nervy*, all three being involved in sensory organ development [19], [23], [24].

**Table 2:** Microarray data confirm differential expression of known SOP genes.

Known SOP genes	Molecular function	SOP/epithelial signal	References
*shaven*	Transcription factor	14,1	[23]
*pebbled* *	Transcription factor	13,5	[18]
*CG32150* *	Protein binding	6,5	[22]
*scabrous*	Signal transduction	6,1	[19]
*nervy*	Transcription factor	6	[24]
*CG32392* *	Microtubule binding	5,9	[22]
*ventral vein lacking*	Transcription factor	5,3	[28]
*miranda* *	Actin binding	5,3	[20]
*senseless*	Transcription factor	4,6	[9]
*phyllopod* *	Ras/MAPK signaling	3,1	[16]
*quail* *	Actin binding	2,8	[22]
*Cytochrome P450-18a1* *	Cytochrome P450	2,7	[22]
*neuralised* *	E3 ubiquitin ligase	2,5	[15]
*seven up*	Transcription factor	2,4	[17]
*cut*	Transcription factor	2,3	[14]
*insensitive* *	Unknown	2,1	[22]
*bancal*	RNA binding	2,1	[27]
*ETS-domain lacking* *	Ras/MAPK signaling	2	[30]
*scratch* *	Transcription factor	2	[29]

Genes previously found to be expressed in SOPs and included in the 127 candidate genes whose expression exhibits a 2.0-fold or greater elevation in SOPs versus epithelial cells.

*Genes also found expressed in proneural clusters according to microarray data obtained by Reeves and Posakony (2005) [22].

## Discussion

In this study, we used laser microdissection to isolate SOPs from the dorsal epithelium of *Drosophila* in order to subsequently analyse the mRNA expression profile. Laser microdissection permits the isolation of single cells from a heterogeneous tissue [25]. The high level of cell homogeneity obtained with this technique permits one to obtain reliable microarray data. In this regard, microdissection, although cumbersome, has certain advantages over other methods of isolating populations of cells such as FACS. In our study, microdisection was applied to tissue freshly dissected and simply fixed in absolute ethanol. This was made possible because the tissue of interest is an epithelium that we are able to dissect from the animal and than flatten. As such, the protocol described here may be adapted to other thin tissues similar to epithelia.

The principal challenge with this technique was to obtain a significant quantity of RNA from SOPs and to ensure that the integrity of the RNA after laser microdissection was sufficient for subsequent gene expression analysis such as quantitative real time PCR and microarrays. Here, we verify the utility and the specificity of the RNA extracted from microdissected SOPs and epithelial cells by performing qRT-PCR on particular genes and undertaking microarray analysis. As expected, we observed by qRT-PCR that *sens*, known to be up regulated in SOPs by proneural protein activity and repressed in non-SOP cells by Notch signaling activation [8], [9], was indeed significantly more expressed in SOPs than in epithelial cells. This result was confirmed by microarray analysis where *sens* was found among the genes exhibiting a two-fold or greater overexpression in SOPs.

Similarly, *gfp*, whose expression was driven specifically in SOPs and their progeny by *neur-*GAL4, was more expressed in microdissected SOPs. However, *gfp* transcripts were still detected in the epithelial sample. This was unexpected since epithelial cells were collected from non-fluorescent areas. It might be possible that a few SOPs, not fluorescent enough to be detectable, were included in epithelial cell selected areas. It might also be possible that there is a weak leak of the *neur-*GAL4 driver onto epithelial cells insufficient to induce a detectable fluorescence.

Unexpectedly, we observed by qRT-PCR and confirmed by microrray, a relatively constant level of *ac* (ratio SOP/epithelial cells  = 0,8 by qRT-PCR and 0,95 by microarray). Indeed, *ac* is a proneural gene whose expression has been shown to be specifically upregulated in proneural clusters and restricted to one cell during SOP specification [3], [4]. However, the expression of *ac* in SOPs has been shown to decrease before cell division [3]. Since we use pupae at 16 h APF, at the moment of SOP first division, we suggest that the relatively similar level of *ac* transcripts observed in SOP and epithelial cells was due to this downregulation phase.

The SOP-enriched genes of the data set obtained in this analysis were classified using Gene Ontology associated terms. This analysis confirmed the specificity of the microdissected SOP samples. Indeed, microdissected SOPs samples were enriched in genes involved specifically in sensory organ development and cell fate related GO terms. Interestingly, eye photoreceptor cell development related GO terms were also enriched in our data. This is not surprising since photoreceptor cells share similar mechanisms of selection with the SOPs including the isolation of one cell among equivalent cluster cells by lateral inhibition mediated by Notch signalling [26]. In this regard, it is interesting that *peb* was highly expressed in SOPs compared to epithelial cells. It has been recently shown that one role of *peb* is to modulate Delta expression during cone cell induction during ommatidial formation [18]. It remains to be known whether *peb* plays a similar role during SOP selection, which it is characterised by an elevated level of Delta.

In accordance with previous studies, many genes (19 out of 127) belonging to the SOP enriched genes identified in our study have been already recognized to be SOP specific. In particular, 11 out of 19 of these known SOP enriched genes are in common with a whole-genome microarray analysis performed with cells belonging to proneural cell clusters [22]. In contrast, some known SOP-specific genes as *sens* and *ct*, were identified in our analysis but not in Reeves and Posakony's study. In their study, proneural cells were sorted by FACS (Fluorescence-Activated Cell Sorting) by using *E(spl)m4*-GFP as proneural cluster-specific marker. As such, the analysis was performed with all cells of proneural clusters including the future SOP. Thus, we expect that some subset of SOP-specific genes also belongs to the genetic profile triggered during proneural cell determination and that another subset is specific for the acquisition of the SOP identity. It is interesting to note that target genes involved in the Notch-mediated lateral inhibition as the *E(spl)* or *bearded (brd)* gene family, which are activated in the future epithelial cells during SOP selection, were either similarly expressed in SOP and epithelial cells or underrepresented in SOPs (for instance, the ratio SOP/epithelial cells for *brd* was 0,35).

Overall, our result confirm the SOP specificity of the gene set identified and we are confident that the approach combining laser microdissected cells and transcriptome analysis will produce exploitable data. Finally, we would like to highlight that a successful characterisation of the transcriptional profile of well-identified precursor cells at a precise moment of development opens multiple possibilities concerning the analysis of the mechanisms underlying precursor cell determination. Thus, the development of a procedure combining laser microdissection and transcriptome analysis represents an undeniably important technical advance for the analysis of biological processes such as fate determination of defined precursor cells.

## Supporting Information

Figure S1 Schematic representation of the procedure. The notum from pupae was manually dissected in PBS, fixed and transferred to a thermolabile membrane slide. The epithelium was facing down membrane. Once dry, the notum, stuck to the membrane, was covered with a slide to maintain the mechanical stability during microdissection. During microdissection the adhesive lid was pressed against the membrane and microdissected cells remained stuck to the lid when the microtube was removed.
(6.86 MB TIF)

Figure S2 qRT-PCR analysis. *Taf11*, *ac*, *gfp*, and *sens* mRNA transcripts from microdissected SOPs and epithelial cells were analysed by qRT-PCR. For each gene, (on the left) PCR amplification curves as function of the number of PCR cycles and (on the right) standard curves, Ct (Cycle threshold) were plotted against serially diluted cDNA samples obtained from aRNA extracted and amplified from 20 whole nota. Note that PCR amplification curves corresponding to SOPs and epithelial cells for *taf11* and *ac* are super-imposed. Ct for SOPs and epithelial cells are similar and data points corresponding to SOPs and epithelial cells cluster together in standard curves (red points). In contrast, PCR amplification curves corresponding to *gfp* and *sens* transcripts are shifted to the left in SOP compared to epithelial cells, showing a stronger expression in SOPs than in epithelial cells. Accordingly two separate groups of data points were observed on the standard curves.
(5.55 MB TIF)
