# Supplementary figures and images for: Laser Microdissection of Sensory Organ Precursor Cells of Drosophila Microchaetes
Source: PLoS One. 2010 Feb 19;5(2):e9285. doi: 10.1371/journal.pone.0009285 (PMC2824816; doi:10.1371/journal.pone.0009285)

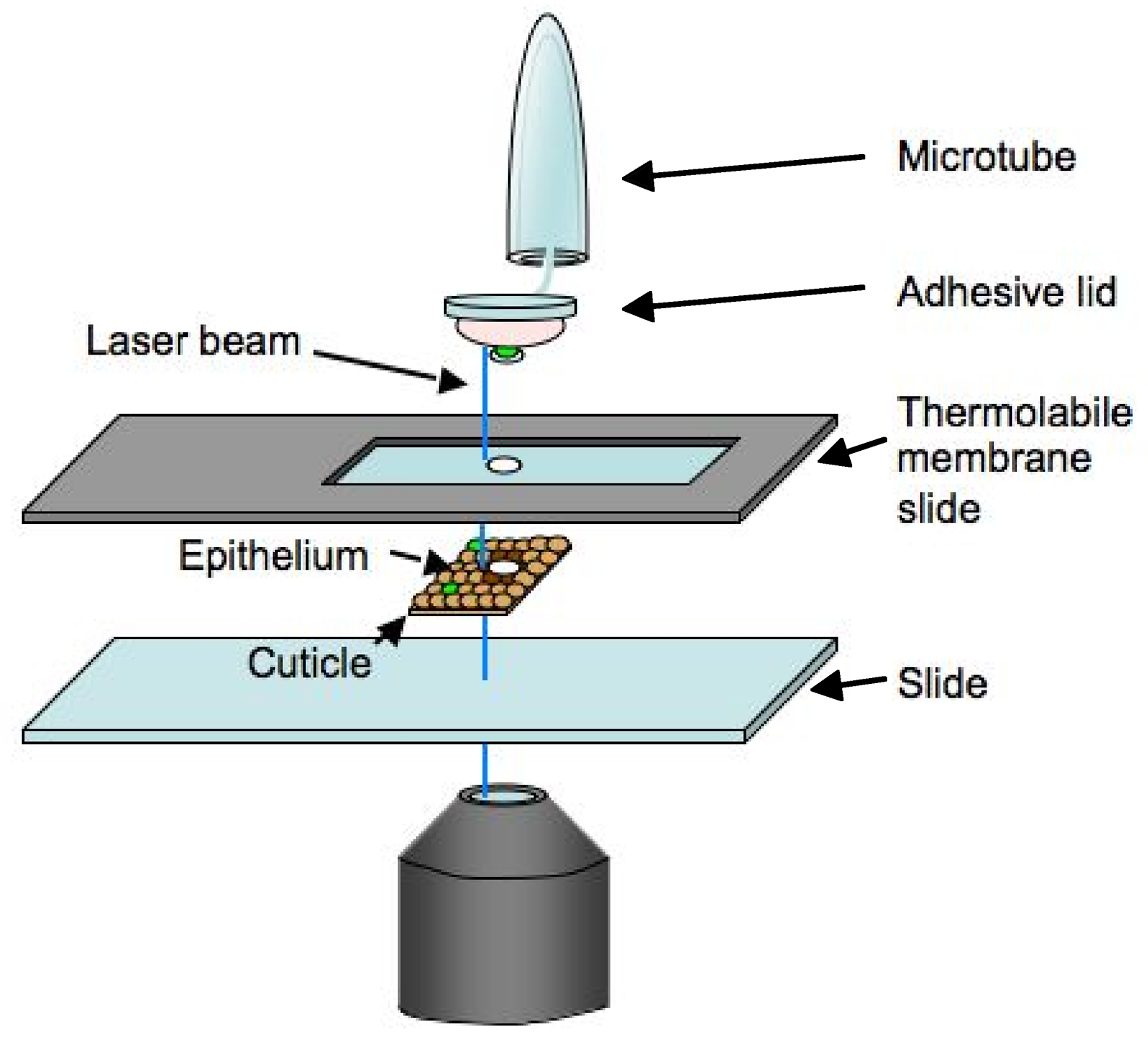

Supplement: Figure S1 — Schematic representation of the procedure. The notum from pupae was manually dissected in PBS, fixed and transferred to a thermolabile membrane slide. The epithelium was facing down membrane. Once dry, the notum, stuck to the membrane, was covered with a slide to maintain the mechanical stability during microdissection. During microdissection the adhesive lid was pressed against the membrane and microdissected cells remained stuck to the lid when the microtube was removed. (6.86 MB TIF) [file pone.0009285.s001.tif]

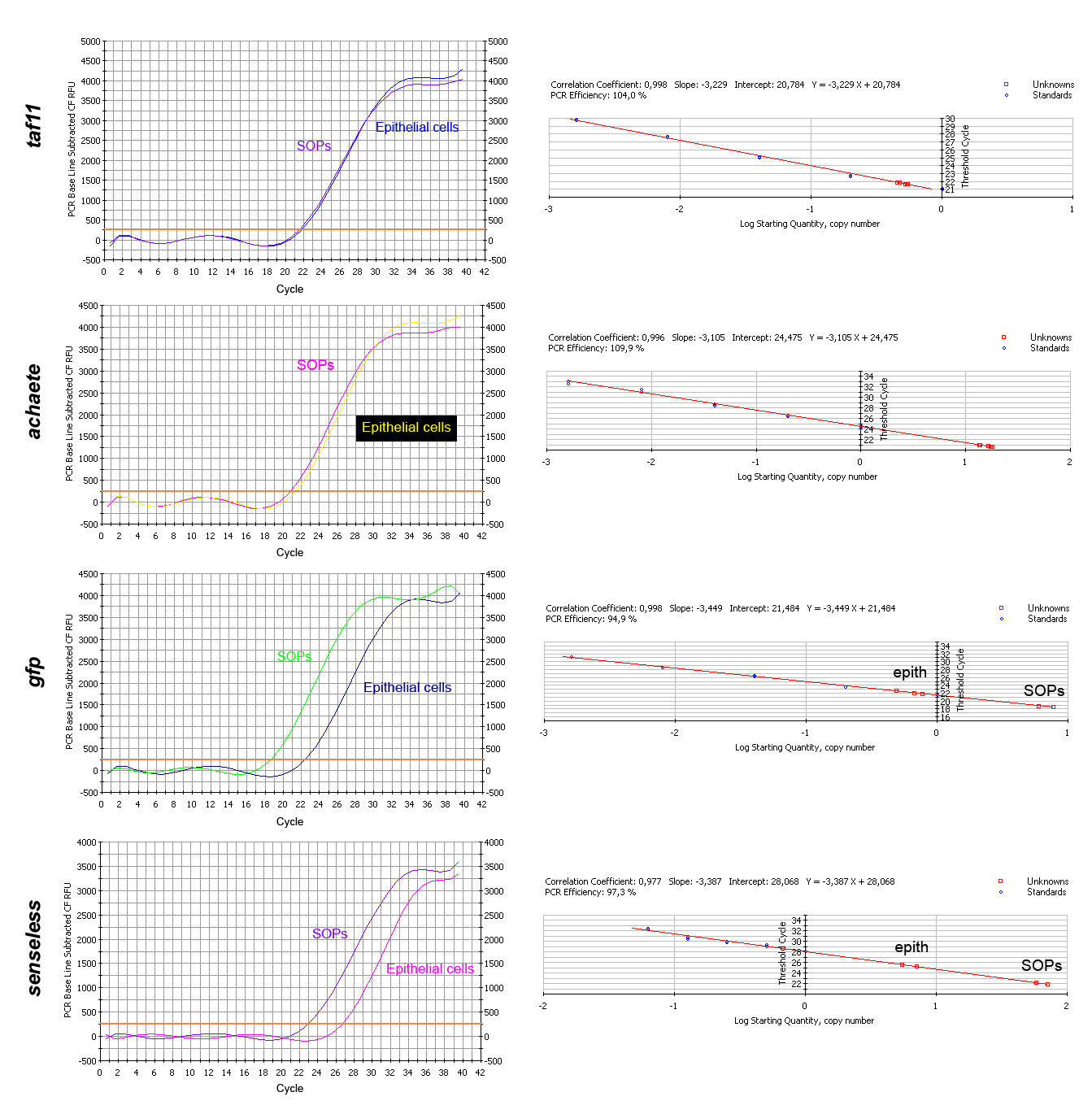

Supplement: Figure S2 — qRT-PCR analysis. Taf11, ac, gfp, and sens mRNA transcripts from microdissected SOPs and epithelial cells were analysed by qRT-PCR. For each gene, (on the left) PCR amplification curves as function of the number of PCR cycles and (on the right) standard curves, Ct (Cycle threshold) were plotted against serially diluted cDNA samples obtained from aRNA extracted and amplified from 20 whole nota. Note that PCR amplification curves corresponding to SOPs and epithelial cells for taf11 and ac are super-imposed. Ct for SOPs and epithelial cells are similar and data points corresponding to SOPs and epithelial cells cluster together in standard curves (red points). In contrast, PCR amplification curves corresponding to gfp and sens transcripts are shifted to the left in SOP compared to epithelial cells, showing a stronger expression in SOPs than in epithelial cells. Accordingly two separate groups of data points were observed on the standard curves. (5.55 MB TIF) [file pone.0009285.s002.tif]
